# Latent class analyses of multimorbidity and all-cause mortality: A prospective study in Chilean adults

**DOI:** 10.1371/journal.pone.0295958

**Published:** 2023-12-19

**Authors:** Gabriela Nazar, Felipe Díaz-Toro, Yeny Concha-Cisternas, Ana María Leiva-Ordoñez, Claudia Troncoso-Pantoja, Carlos Celis-Morales, Fanny Petermann-Rocha

**Affiliations:** 1 Departmento de Psicología, Universidad de Concepción, Concepción, Chile; 2 Facultad de Enfermería, Universidad Andres Bello, Santiago, Chile; 3 Escuela de Kinesiología, Facultad de Salud, Universidad Santo Tomás, Santiago, Chile; 4 Pedagogía en Educación Física, Facultad de Educación, Universidad Autónoma de Chile, Providencia, Chile; 5 Instituto Anatomía, Histología y Patología, Facultad de Medicina, Universidad Austral de Chile, Valdivia, Chile; 6 Centro de Investigación en Educación y Desarrollo (CIEDE-UCSC), Departamento de Salud Pública, Facultad de Medicina, Universidad Católica de la Santísima Concepción, Concepción, Chile; 7 School of Cardiovascular and Metabolic Health, University of Glasgow, Glasgow, United Kingdom; 8 Human Performance Laboratory, Education, Physical Activity and Health Research Unit, Universidad Católica del Maule, Talca, Chile; 9 Centro de Investigación Biomédica, Facultad de Medicina, Universidad Diego Portales, Santiago, Chile; Hospital de la Santa Creu i Sant Pau, SPAIN

## Abstract

Multimorbidity patterns can lead to differential risks for all-cause mortality. Within the Chilean context, research on morbidity and mortality predominantly emphasizes individual diseases or combinations thereof, rather than specific disease clusters. This study aimed to identify multimorbidity patterns, along with their associations with mortality, within a representative sample of the Chilean population. 3,701 participants aged ≥18 from the Chilean National Health Survey 2009–2010 were included in this prospective study. Multimorbidity patterns were identified from 16 chronic conditions and then classified using latent class analyses. All-cause mortality data were extracted from the Chilean Civil Registry. The association of classes with all-cause mortality was carried out using Cox proportional regression models, adjusting by sociodemographic and lifestyle variables. Three classes were identified: a) Class 1, the healthiest (72.1%); b) Class 2, the depression/cardiovascular disease/cancer class (17.5%); and c) Class 3, hypertension/chronic kidney disease class (10.4%). Classes 2 and 3 showed higher mortality risk than the healthiest class. After adjusting, Class 2 showed 45% higher mortality risk, and Class 3 98% higher mortality risk, compared with the healthiest class. Hypertension appeared to be a critical underlying factor of all-cause morbidity. Particular combinations of chronic diseases have a higher excess risk of mortality than others.

## Introduction

Chronic diseases are responsible for nearly 41 million deaths annually, equivalent to 74% of all deaths on the planet. Of all these deaths, 77% are in low-middle-income countries (LMICs) [1]. Although the burden of single chronic diseases is high, it is becoming highly prevalent among the adult population the coexistence of two or more chronic diseases [2].

Multimorbidity refers to the simultaneous presence of two or more chronic diseases within an individual, which may or may not share a common etiology [3, 4]. This condition often results in polypharmacy, reduced functionality, and a decline in quality of life and overall health [5]. Furthermore, multimorbidity influences the utilization of clinical services and healthcare consultations [6], thereby elevating the disease burden and healthcare costs [7]. Existing evidence suggests that the prevalence of multimorbidity varies between 30% and 40% [8]. In Chile, 34% of adults aged ≥15 have two to four chronic diseases [9]. The relationship between chronic disease and mortality is well-known; however, the co-occurrence of specific conditions can be more detrimental than the combination of others; thus, the natural clustering of chronic conditions might contribute to the differential influence of chronic conditions on all-cause mortality. Most evidence about multimorbidity patterns recognizes common groupings. Among them, there are combinations of cardiovascular (CVD) and metabolic diseases [10, 11], mental health problems [10, 11], respiratory multimorbidity [12], and musculoskeletal disorders [13].

Multimorbidity is closely related to mortality, and research suggests that the presence of 3 or 4 diseases increases the risk of mortality, a figure that increases exponentially in people with more diseases compared to patients who do not have chronic diseases [14].

Additionally, some particular groupings of diseases can have a higher incidence of mortality. Research identifies heart disease patterns as a group with a higher mortality hazard than respiratory or musculoskeletal classes [15].

Although mortality is considered a core outcome in multimorbidity research [16], this relationship has barely been addressed in LMICs [17]. Much of the evidence has been derived from high-income countries [18, 19].

In Chile, studies tend to focus on single diseases or comorbid pairs but commonly do not address patterns of multimorbidity or any particular combination of diseases and their relationship with mortality [11]. Moreover, patterns might be specific to LMICs due to particular cultural and socioeconomic factors, nutritional transition and health behavior, as well as access to health care [13].

Based on the above, this study sought to i) identify multimorbidity patterns of chronic disease and ii) determine their association with all-cause mortality in a representative sample of the Chilean adult population.

## Material and methods

### Study design

This longitudinal study used data from the Chilean National Health Survey (CNHS) conducted between 2009 and 2010 [20]. The CNHS is the largest, nationally representative population-based health survey. It is a stratified, multistage probability sample of Chilean residents ≥ 15 years old [20]. It assesses health biomarkers (e.g., blood pressure, cholesterol, uremia, glycosylated hemoglobin), health conditions and morbidities (e.g., diagnoses of chronic diseases, cognitive impairment), lifestyle (e.g. physical activity, diet and eating behavior), health risk factors (e.g. obesity, alcohol consumption, smoking habits) and psychosocial variables (e.g., social support, group membership). Data were collected by trained professionals using standardized protocols.

The CNHS 2009–2010 was funded by the Chilean Ministry of Health and approved by the Ethics Research Committee of the School of Medicine at the Pontificia Universidad Católica de Chile. All participants provided written consent before participation [20]. All data are freely available at the following link http://epi.minsal.cl/bases-de-datos/.

From the total CHNS 2009–2010 (n = 5,293) and after excluding participants with missing data (n = 1,592), the final sample of this study totaled 3,701 individuals, 59.5% women, mean age of 47.2 years (S1 Fig). No differences in terms of sex were discerned among individuals who were not included in the study (59.1% women, p = 0.441). Nonetheless, those individuals who did not participate due to missing data were found to be younger by three years, with an average age of 44.5 (p = 0.003).

### Outcome

The outcome was all-cause mortality. The date of death was obtained from death certificates linked to the Chilean Civil Registry and Identification. Mortality data were collected until the 31^st^ of December 2020. Therefore, mortality follow-up was censored on this date or the date of death in case it occurred earlier.

### Chronic conditions

All participants self-reported up to 20 chronic diseases that were medically diagnosed at the baseline. Trained nurses asked: “Has a doctor, nurse, or another health professional ever told you that you have or have had (name of the disease)?” Of the chronic conditions asked, those with a higher prevalence (greater than 2%) and associated with lifestyle were included in this analysis (16 in total): hypertension, diabetes, high cholesterol, stroke, peripheral vascular disease, acute myocardial infarction, arthritis, arthrosis, colon cancer, gastric cancer, gallbladder cancer, chronic kidney disease, asthma, cataracts, angina, and depression.

### Covariates

#### Sociodemographic variables

Age (in years), sex (women or men), place of residence (urban or rural), and educational level (low: < 8 years, middle: 8–12 years, or high: >12 years) were self-reported using standardized questionnaires.

#### Lifestyle and nutritional status

Smoking, alcohol consumption, physical activity (PA), sitting time, fruit and vegetable intake, and body mass index (BMI) were treated as covariates. Smoking was self-reported and classified as no smoker, regular smoker, occasional smoker, and ex-smoker according to the frequency of tobacco consumption. Alcohol consumption was assessed by the Alcohol Use Disorders Identification Test, AUDIT [21], and classified as no-risk (score < 8) or risk use (score ≥ 8). Total PA was assessed using the Global Physical Activity Questionnaire [22] informed as MET/min/week. Physical inactivity was defined as PA <600 METs/min/week or equivalent. Sitting time was defined as sitting or reclining at work or home, e.g., sitting at a desk, traveling by car, bus, or train, reading, playing cards, or watching TV, but it did not include sleep time. It was estimated by the following question: “How much time do you usually spend sitting or reclining on a typical day?” The answers were classified in tertiles according to the number of h/day in sedentary activities. Dietary intake of fruits and vegetables was assessed with the following questions: ‘In a typical/ordinary week, how many days do you eat fruit?’ and ‘In a typical/ordinary week, how many days do you eat vegetables?’ which was then converted into grams and classified according to the accomplishment (yes or no) of the recommendation of daily intake of five portions of fruits and vegetables. Nutritional status was assessed by standardized protocols [20] using the body mass index (BMI: kg/m^2^). The WHO criteria for adults was used to define underweight (<18.5 kg/m^2^), normal (18.5 to 24.9 kg/m^2^), overweight (25.0 to 29.9 kg/m^2^) and obese (≥30.0 kg/m^2^) [23].

### Statistical analyses

The baseline characteristics of participants are presented as mean and standard deviation (SD) for continuous variables and percentages with their 95% confidence intervals (CI) for categorical variables by multimorbidity classes.

Latent class analysis (LCA) was performed considering the total number of participants with available data (3,701). The optimal number of latent classes was determined using the adjusted Bayes Information Criterion (BIC) and the consistent Akaike Information Criterion (AIC) in Stata (S1 Table). Five final converged models were compared. The goodness-of-fit statistic for each latent class model (i.e., the model fit is to compare the model we fit with a saturated model) was tested using the likelihood-ratio G2 test. As none of the models fit worse than the saturated model, the model with the lowest BIC was finally used to create the classes (Model 3, S1 Table). We used this criterion considering that the AIC has a higher false positive rate than the BIC (i.e., it is more likely to wrongly recommend a higher number of classes than reality).

Univariate multinomial logistic regression models assessed the association of each multimorbidity class with sociodemographic and lifestyle behavior. Results were expressed in OR and their 95% confidence intervals (CI). To investigate the association between multimorbidity classes and all-cause mortality, Cox proportional regression models were performed. The results were reported as hazard ratio (HR) with their respective 95% CI. Class 1, the healthiest class, was used as the reference for all analyses.

Analyses were run using four models: Model 1 was unadjusted. Model 2 was adjusted by age, sex, educational level, and zone of residence. Model 3 was additionally adjusted by smoking, alcohol, PA, consumption of fruits and vegetables, and sitting time. Model 4 was as per model 3 but had additional BMI.

All statistical analyses were conducted using Stata V18 software (StataCorp; College Station, TX) and). A *p-*value below 0.05 was considered statistically significant.

## Results

Each participant was assigned to one class after selecting the best latent class prediction model (S1 Table). The latent classes were labeled according to the chronic conditions that were more prevalent in each group as follows: a) Class 1, healthiest class; b) Class 2, depression/CVD/cancer disease class; and c) Class 3, hypertension/chronic kidney disease class (S2 Fig).

Table 1 shows the prevalence of chronic disease in the general population and for the three multimorbidity classes. Table 2 presents the baseline characteristics of participants assigned to each class. From the total sample (n = 3,701), 2,669 participants (72.1%) belong to Class 1, described as the ’healthiest’. The prevalence of all health conditions assessed in this class was lower than in the other two classes (Table 1). This group showed the lowest mean of age (41.2 years [15.2]), a higher proportion of participants with low educational level (18.1% >12 years of schooling), the lowest BMI (27.5 kg/m^2^), and the highest proportion of current smokers (40.1%) (Table 2). Class 2 accounts for 17.5% of the total sample and was named the ’depression/CVD/ cancer class.’ Compared to the other two classes, it showed a higher prevalence of depression (53.1%), high cholesterol (47%), acute myocardial infarction (40.4%), angina (45.6%), stroke (36.1%), diabetes type II (21.1%), peripheral vascular disease (16.2%), gastric cancer (23.9%), and gallbladder cancer (15.3%). Although the prevalence of hypertension (48.2%) was high, it was lower than Class 3 (Table 1). Participants in Class 2 had a mean age of 62.2 years [13.2]) and were mainly women from urban areas (Table 2). Participants in Class 2 also showed the lowest proportion of current smokers. Class 3 had the highest prevalence of hypertension (79.8%), chronic kidney disease (29.6%) and cataracts (21.6). It included 384 participants (10.4%) and was labeled as ’hypertension/chronic kidney disease class.’ The main characteristic of this class is the highest prevalence of hypertension (79.8%) and chronic kidney disease (29.6%). Except for these conditions, Class 3 showed a similar profile to Class 2 but less accentuated, including conditions such as high cholesterol, diabetes, peripheral vascular disease and myocardial infarction. This class was composed mainly of women (77.8%) and had the lowest percentage of people with low educational levels.

**Table 1:** Prevalence of chronic disease in the general population and for each class.

	Prevalence (%)			
Chronic Disease	General population	Class 1	Class 2	Class 3
		The healthiest class	Depression/CVD /cancer class	Hypertension/chronic kidney class
Hypertension	31.7	14.5	48.2	79.8
Diabetes	7.9	1.1	21.1	22.7
High colesterol	21.9	12	47	39.4
Stroke	14.2	9.6	36.1	15.7
Peripheral vascular disease	6.5	3.4	16.2	10.3
Arthritis	3.5	0.7	11.4	7.7
Acute Myocardial infarction	20.7	15.2	40.4	26.7
Colon cancer	3.9	3.4	8.4	2.6
Gastric cancer	12.2	10.1	23.9	11.5
Depression	16.1	12.7	53.1	2.9
Chronic kidney disease	7.3	0.9	7.3	29.6
Gallbladder cancer	5.7	4.4	15.3	3.7
Asthma	10	6.1	33.5	7.8
Cataracts	6.3	0.8	11.1	21.6
Angina	14.5	9.6	45.6	10.2
Arthrosis	6.2	1.4	20.9	12.9

**Table 2:** Baseline characteristics of the three multimorbidity classes.

	Class 1	Class 2	Class 3
	N = 2,669	N = 648	N = 384
	72.1%	17.5%	10.4%
**Age, mean (SD)**	41.2 (15.2)	66.2 (13.2)	56.4 (12.9)
**Sex, % (95% CI)**			
Women	56.5 (54.6–58.3)	60.9 (57.1–64.6)	77.8 (73.4–81.7)
Men	43.4 (41.6–45.3)	39.1 (35.3–42.8)	22.2 (18.2–26.5)
**Educational level, % (95% CI)**			
Low (< 8 years)	23.8 (22.5–25.4)	12.1 (9.7–14.7)	9.3 (6.8–12.7)
Middle (8–12 years)	58.1 (56.2–59.9)	39.2 (35.5–43,1)	46.6 (41.6–51.6)
High (>12 years)	18.1 (16.6–19.5)	48.7 (44.9–52.6)	44.1 (39.1–49.1)
**Place of residence, % (95% CI)**			
Urban	85.3 (83.9–86.6)	83.6 (80.5–86.2)	85.1 (81.2–88.3)
Rural	14.6 (13.4–16.1)	16.3 (13.7–19.4)	14.8 (11.6–18.7)
**Smoking % (95% CI)**			
Never	37.6 (35.7–39.4)	52.3 (48.4–56.1)	36.2 (31.5–41.1)
Previous	22.3 (20.8–23.9)	29.6 (26.2–33.2)	32.1 (27.5–36.8)
Current	40.1 (38.1–41.8)	18.1 (15.2–21.2)	31.7 (27.3–36.5)
**AUDIT Score, % (95% CI)**			
Low risk	89.4 (88.2–90.6)	93.8 (91.7–95.4)	94.1 (91.1–95.9)
Moderate risk	8.3 (7.4–9.5)	5.4 (3.9–7.4)	3.9 (2.3–6.4)
High risk	2.3 (0.7–3.5)	0.8 (0.2–2.1)	2 (0.4–2.9)
**BMI (kg/m** ^**2**^ **), mean (SD)**	27.5 (4.9)	28.5 (5.5)	30.6 (5.5)
**BMI categories, % (95% CI)**			
Underweight	1.4 (1.1–1.9)	1.7 (1.1–2.6)	1.8 (1.2–2.8)
Normal weight	27.8 (26.4–29.2)	28.8 (26.2–31.6)	35.6 (32.7–38.6)
Overweight	40.9 (39.3–42.5)	42.2 (39.2–45.1)	42.5 (39.545.5)
Obese	29.9 (25.7–32.1)	27.3 (22.7–28.8)	20.1 (16.6–21.4)
**Physical Activity, % (95% CI)**			
Active	68.5 (67.2–70.1)	73.2 (68.2–73.7)	73.2 (69.2–74.8)
Inactive	31.5 (29.8–32.8)	22.8 (26.3–31.8)	26.8 (25.2–30.7)
**Recommended consumption of fruits and vegetables, % (95% CI)**			
Yes	65.1 (62.5–66.1)	68.9 (66.4–74.1)	68.2 (64.3–72.6)
No	34.9 (33.8–37.5)	31.1 (27.5–35.3)	31.8 (25.4–33.5)
**Sitting time, % (95% CI)**			
Tertile 1	46.5 (44.6–48.4)	39.9 (36.1–43.8)	45 (40.4 49.6)
Tertile 2	26.8 (25.1–28.5)	31.4 (27.8–35.2)	30.5 (26.3–34.9)
Tertile 3	26.7 (25.1–28.3)	28.7 (25.2–32.5)	24.5 (20.7–28.7)
**Number of comorbidities (mean, SD)**	1.06 (0.9)	3.4 (1.2)	5.03 (1.5)

SD: standard deviation; 95% CI: 95% confidence intervals.

Table 3 shows the logistic regression model for the latent classes. We observed that older participants had a higher likelihood of belonging to the classes with the highest prevalence of multimorbidity, specifically for each year of age, the likelihood of belonging to Class 2 increased by 11% (OR:1.11 [95% CI: 1.10–1.13]), and Class 3, 6% (OR: 1.06 [95% CI:1.05–1.08]). Compared to males, female participants showed a higher probability of belonging to Class 2 (OR:1.20 [95% CI: 1.001–1.43]) and Class 3 (OR:2.70 [95% CI: 2.10–3.48]) and participants with middle and high years of schooling had a higher probability of belonging to Class 1 than those with the lowest educational level. Moreover, participants who were current smokers showed a lower probability of belonging to Class 2 (OR:0.34 [95% CI: 0.26–0.43]) and Class 3 than previous smokers (OR:0.55 [95% CI: 0.42–0.72]), and those who reported a moderate risk of alcohol consumption had a lower probability of belonging to Class 2 (OR:0.61 [95% CI: 0.42–0.88]) and Class 3 (OR:0.44 [95% CI: 0.25–0.75]) than those with low-risk alcohol consumption. Participants classified as active showed a lower likelihood of belonging to Classes 2 (OR:0.53 [95% CI: 0.44–0.63]) and 3 (OR:0.80 [95% CI: 0.64–0.99]) compared with inactive participants (Table 3).

**Table 3:** Odds ratios multinomial regression model about the latent class on demographic variables and health behavior.

	Class 2 vs. Class 1		Class 3 vs. Class 1	
	OR (95% CI)	p-value	OR (95% CI)	p-value
**Age (continuos)**	1.11 (1.10–1.13)	**<0.01**	1.06 (1.05–1.08)	**<0.01**
**Sex**				
Men	1.00 (ref.)		1.00 (ref.)	
Women	1.20 (1.001–1.43)	**0.04**	2.70 (2.10–3.48)	**<0.01**
**Educational level (%)**				
Low < 8 years	1.00 (ref.)		1.00 (ref.)	
Middle 8–12 years	0.24 (0.20–0.30)	**<0.01**	0.33 (0.26–0.41)	**<0.01**
High >12 years	0.18 (0.14–0.24)	**<0.01**	0.25 (0.21–0.30)	**<0.01**
**Place of residence**				
Urban	1.00 (ref.)		1.00 (ref.)	
Rural	0.88 (0.69–1.11)	0.29	0.98 (0.73–1.33)	0.93
**Smoking**				
Never	1.04 (0.85–1.28)	0.64	0.67 (0.51–0.87)	**0.03**
Previous	1.00 (ref.)		1.00 (ref.)	
Current	0.34 (0.26–0.43)	**<0.01**	0.55 (0.42–0.72)	**<0.01**
**AUDIT Score**				
Low risk	1.00 (ref.)		1.00 (ref.)	
Moderate risk	0.61 (0.42–0.88)	**<0.01**	0.44 (0.25–0.75)	**0.03**
High risk	0.71 (0.21–2.33)	0.58	0.58 (0.17–1.09)	0.37
**Physical Activity**				
Active	0.53 (0.44–0.63)	**<0.01**	0.80 (0.64–0.99)	**0.05**
Inactive	1.00 (ref.)		1.00 (ref.)	
**Recommended consumption of fruits and vegetables**				
Yes	0.86 (0.68–1.10)	0.24	0.82 (0.65–1.05)	0.12
No	1.00 (ref.)		1.00 (ref.)	
**Sitting time**				
Tertile 1	1 (ref.)		1.00 (ref.)	
Tertile 2	1.30 (1.06–1.58)	**0.01**	0.81 (0.62–1.06)	0.13
Tertile 3	1.05 (0.78–1.42)	0.71	0.89 (0.61–1.29)	0.55

Results are expressed as odds ratios (OR) and their 95% confidence intervals (CI). Class 1 was used as the reference group.

The analysis of the association between each latent class and all-cause mortality is shown in Table 4. Over 10.9 follow-up years (interquartile range: 10.7 to 11.8), 407 people died (10.9%). For the unadjusted model, the risk of all-cause mortality was higher in Class 2 ’depression/CVD /cancer class’ (HR: 7.68 [95% CI:6.15–9.61]) and Class 3 ’Hypertension and chronic kidney disease class (HR: 4.46 [95% CI: 3.35–5.94]) compared with the reference class, Class 1 or the healthiest. After adjusting for sociodemographic variables, the observed risk was attenuated but remained significant (model 2): Class 2 (HR: 1.54 [95% CI: 1.20–1.98]) and Class 3 (HR: 2.06 [95% CI: 1.53–2.77]). After adding lifestyle behavior to model 2 (model 3), Class 2 showed higher mortality risk compared to the healthiest class (HR:1.47 [95% CI: 1.13–1.91]), and the same occurred for Class 3 (HR:1.85 [95% CI: 1.36–2.52]). In Model 4, which added BMI as a confounder, Classes 2 and 3 showed an increased risk of mortality compared to Class 1, being 45% for Class 2 (HR:1.45 [95% CI: 1.13–1.90]) and 98% for Class 3 (HR:1.98 [95% CI: 1.45–2.70]).

**Table 4:** Association between the created classes and all-cause mortality.

N = 3,701	Model 1	*p-value*	Model 2	*p-value*	Model 3	*p-value*	Model 4	*p-value*
	HR (95%CI)		HR (95%CI)		HR (95%CI)		HR (95%CI)	
**Class 1**	1.00 (ref.)		1.00 (ref.)		1.00 (ref.)		1.00 (ref.)	
**Class 2**	**7.68 (6.15–9.61)**	<0.01	**1.54 (1.20–1.98)**	<0.01	**1.47 (1.13–1.91)**	0.03	1**.45 (1.13–1.90)**	0.04
**Class 3**	**4.46 (3.35–5.94)**	<0.01	**2.06 (1.53–2.77)**	<0.01	**1.85 (1.36–2.52)**	<0.01	**1.98 (1.45–2.70)**	<0.01

Data presented as Hazard Ratios (HR) and their 95% CI. Individuals in class 1 were used as the reference. Model 1 was unadjusted. Model 2 was adjusted by age, sex, educational level, and zone of residence. Model 3 was additionally adjusted by smoking, alcohol, physical activity, consumption of fruits and vegetables, and sitting time. Model 4 was as per model 3 but additionally body mass index.

**Fig 1:**
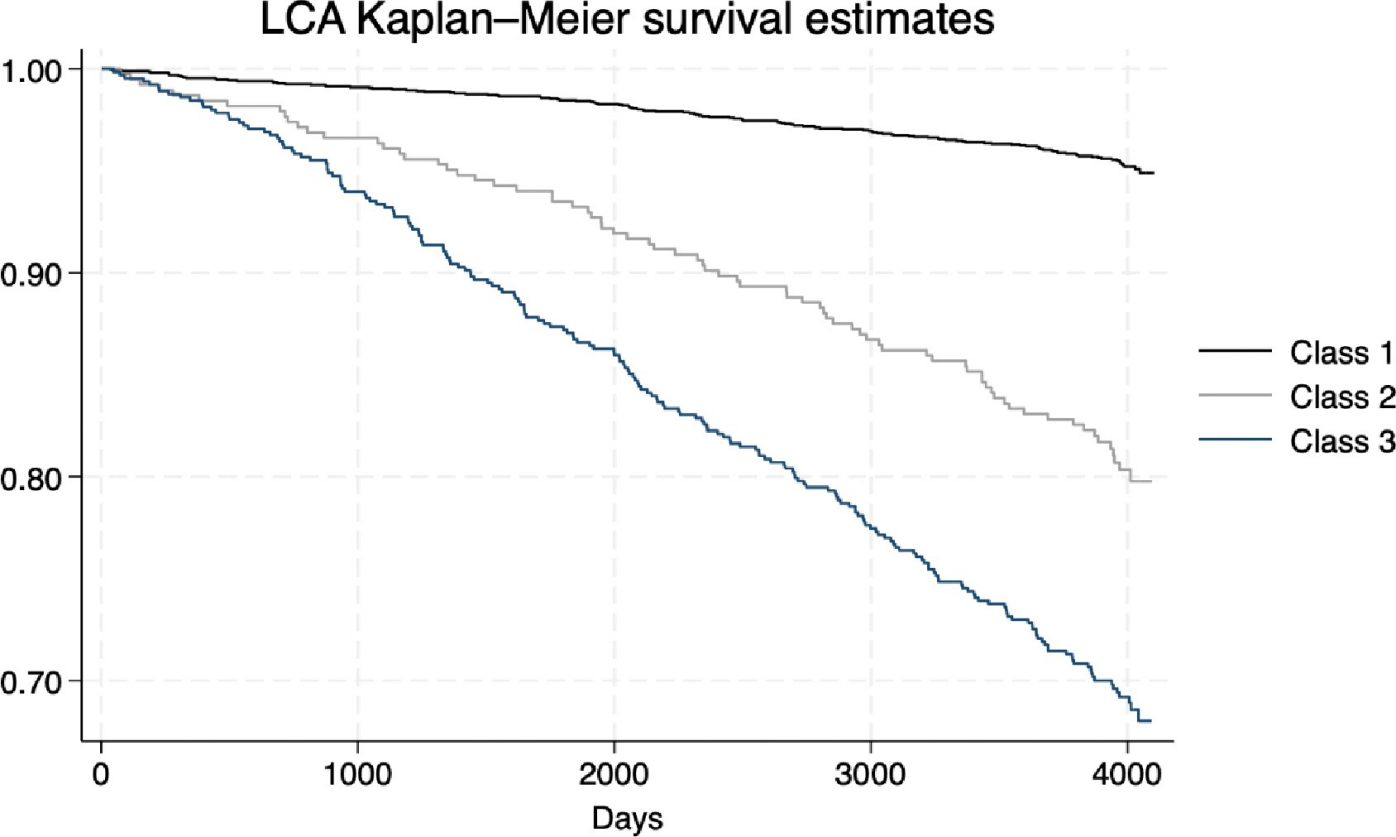
Kaplan-Meir survival estimates.

Kaplan–Meier survival estimates by classes for the general population are shown in Fig 1. Briefly, for Class 3, lower survival rates compared to Class 1 were observed (log-rank < 0.001).

## Discussion

### Main findings

Through LCA, three distinct classes were identified: Class 1, designated as the ’healthiest group’ (72.1%); Class 2, termed the ’depression/CVD/cancer class’ (17.5%); and Class 3, characterized as the ’hypertension/chronic kidney disease class’ (10.4%). The probability of falling into multimorbidity classes (2 and 3) was observed to be higher among females and exhibited an increase with each successive year of age. Participants in Class 2 had 45% more risk of dying than those in the healthiest class, and this risk increased to 98% for the Class 3 group. In this context, patterns of morbidities characterized by cardiovascular conditions, particularly hypertension, were critical factors leading to mortality.

### What is already known on this topic

The evidence indicates that multimorbidity is not a random occurrence; rather, it involves the simultaneous presence of diseases that can be structured into patterns. A diverse range of chronic condition patterns has been delineated in the literature using various analytical approaches, including factor analysis [24], cluster analysis [25], and LCA [26]. Despite the diversity in statistical methods for identifying multimorbidity patterns, authors consistently report similar findings across these analyses [11].

Patterns vary depending on the number and types of health conditions assessed [27]; however, most literature identifies close to three groups. The review of Ng et al. identified a combination of cardiometabolic disease, mental health problems and allergic diseases [27]. A review of multimorbidity in 14 studies identified three common groupings in 10 out of the 14 studies analyzed [11]: the cardiovascular/metabolic pattern, with high blood pressure and diabetes as the most common conditions; the mental health group; and musculoskeletal conditions group. Another study using data from nine countries worldwide, and 41,909 noninstitutionalized adults older than 50 years, identified two or three multimorbidity patterns per country with a variety of patterns across several countries: cardio-respiratory (angina, asthma, and chronic obstructive pulmonary disease), metabolic (diabetes, obesity, and hypertension), and mental- articular patterns (arthritis and depression) [28].

Using LCA, Bayes et al. identified three classes in three population-based studies in LMICs: ’cardiometabolic,’ ’respiratory- mental- articular,’ and ’healthy’ [13]. The study of Olaya et al., 2017 –in a representative sample of 4753 Spanish aged 50 and above–which assessed 11 chronic conditions, also identified three classes named the ’healthy class,’ ’cardiorespiratory/mental/ arthritis class’, and the ’metabolic/stroke class’ [26]. Also, using LCA, a study in the Korean population identified three classes: the ’relatively healthy group’ (60.4% of the population); the ’cardiometabolic conditions’ group (27.8%); and the ’arthritis, asthma, allergic rhinitis, depression, and thyroid disease’ group (11.8%) [29]. Similar to our results, previous studies identify a healthy or minimal disease class, which tends to be the largest group [13, 26, 29].

A prevalent multimorbidity pattern frequently identified in the literature is the cardiovascular and metabolic cluster, consistently emerging across various analytical methodologies. A recent review of 39 articles noted that the most prevalent multimorbidity grouping encompassed cardiometabolic and cardiorespiratory conditions [30].

As per our study findings, hypertension emerged as the most prevalent condition not only within the healthiest class but also in the two classes characterized by a more substantial burden of morbidity. These results align with those of a study involving 2143 older Brazilian adults [31]. An additional study, utilizing exploratory factor analysis data collected from countries with varying income levels, demonstrated hypertension to be the most predominant condition among distinct classes [28]. Furthermore, a study involving 4127 elderly individuals in Germany unveiled that combinations such as hypertension and diabetes, along with hypertension and stroke, frequently manifested regardless of age, gender, and other health conditions [24].

Our analyses have revealed that Classes 2 and 3 exhibit significantly elevated mortality risks compared to the healthiest class. Of particular note, depression exhibited a notably high prevalence within Class 2, surpassing that of the general population and the other two classes. One plausible explanation is that Class 2 demonstrated a heightened incidence of diverse ailments, with multimorbidity closely intertwined with depression. A study conducted on the Chilean population found that the presence of ≥2 chronic diseases and low self-rated health posed a risk for depression [32]. Conversely, health conditions prevalent within Class 2, such as cancer, acute myocardial infarction, and stroke, have been linked to depression and mood disorders [33, 34]. The correlation between health conditions and depression can be attributed to distinct physiological and psychosocial factors, including inflammatory processes, functional impairments resulting from the disease, and the consequent declines in quality of life and independence.

Hypertension was the most prevalent common condition in Classes 2 and 3, followed by high cholesterol. Accordingly, in a Brazilian study on older adults, the highest mortality rate was observed in the combination of high blood pressure with other conditions [31]. A potential explanation for our results is based on the burden of hypertension and CVD. Our study suggests that hypertension could be an underlying factor in multiple chronic diseases and confirms that it is related to serious health outcomes, including mortality. Moreover, international reports have informed that CVD, a composite of cardiovascular death, myocardial infarction, stroke, and heart failure, is the leading cause of mortality worldwide [35, 36], with more than 70% of the deaths in LMICs [37]. Metabolic factors were the predominant risk factors for cardiovascular disease, with hypertension being the largest [38]. Moreover, hypertension is considered one of the most important preventable causes of premature death and cardiovascular disease globally [39] and it was associated with a higher risk for CVD and all-cause mortality, with stronger associations with a younger age of onset [35].

High cholesterol was another highly prevalent condition in the two morbidity classes identified. Abnormal lipid metabolism and elevated blood pressure are cardiometabolic risk factors that can synergistically affect cardiovascular diseases and all-cause mortality [40].

### What this study adds

This study identified distinct classes based on 16 chronic diseases, uncovering an elevated all-cause mortality risk within classes characterized by a high prevalence of multimorbidity. Notably, hypertension emerged as the prevailing health condition across various morbidity classes, particularly within those classes exhibiting the highest mortality risk.

To the best of our knowledge, this is the first research to analyze the association between patterns of chronic conditions, as determined by LCA, and mortality within a representative sample from Chile. As a middle-income nation undergoing accelerated aging, this country’s demographic landscape is shifting. Given that age constitutes a pivotal factor in developing chronic diseases, a surge in the number of individuals grappling with multiple chronic conditions is anticipated. These risk groups encompass not only older adults but also women, underscoring the imperative to address multimorbidity by tailoring interventions to the specific needs of these more vulnerable cohorts.

The results also highlight the need to recognize multimorbidity not as an aggregation of isolated conditions, but as an integrated and complex cluster of diseases that interact with each other. Hypertension appears as a critical factor in different patterns of multimorbidity. This is particularly important since cardiometabolic risk factors are highly prevalent in Chile. In the CNHS 2016–2017, almost 30% of the population aged ≥15 was diagnosed with hypertension, 27.8% had high total cholesterol, 74.4% presented overweight or obesity and 33% were current smokers [41]. Moreover, the prevalence of metabolic syndrome reached 41.2% with an increase of 18% from 2010 [42].

The relationship between the cardiometabolic pattern of diseases and mortality places health promotion and prevention initiatives as key strategies for the healthcare system. LMICs face the need to decrease the burden of CVD, which includes the promotion of healthy lifestyles across the life course, early screening of risk factors, and the need to guarantee access, opportunity, and quality of health services.

### Limitations

We acknowledge some important limitations. Firstly, the use of self-report measures to access data on morbidities might lack accuracy. Additionally, the number and types of classes identified in this study were influenced by the health conditions available. Secondly, our study focuses on the adult population, whereas most studies examining multimorbidity patterns have been conducted in older populations. This can make it more complex to compare results, as the prevalence of chronic conditions is influenced by age. Thirdly, another limitation stems from the assessment of morbidity only at baseline, so there is no information about changes in chronic conditions over time. The analysis did not include data on the age of onset of the chronic condition, despite its importance, as earlier onset can lead to a more complex illness evolution and higher mortality rates [43]. Finally, as per any observational study, causality cannot be inferred.

## Conclusion

Chronic conditions within the Chilean population can be systematically categorized into distinct classes, with specific combinations of morbidities yielding more adverse impacts than others. Notably, hypertension emerged as a recurrent factor underpinning Classes characterized by an elevated mortality risk. With the prevalence of multimorbidity reaching significant levels and a wide array of condition patterns evident, there arises a necessity to approach healthcare from a person-centered perspective rather than a single disease-focused approach.

## Supporting information

S1 Table Comparison between models n = 3,701. (DOCX)

S1 Fig Participants included in the analysis (electronic version). (DOCX)

S2 Fig Latent classes identified. (DOCX)
