# Supplementary material for: Latent class analyses of multimorbidity and all-cause mortality: A prospective study in Chilean adults
Source: PLoS One. 2023 Dec 19;18(12):e0295958. doi: 10.1371/journal.pone.0295958 (PMC10729966; doi:10.1371/journal.pone.0295958)
Supplement: S1 Table — (DOCX) [file pone.0295958.s001.docx]

# **S1 Table. Comparison between models n=3,701**

| **Number of latent classes** | **AIC** | **BIC** | **Likelihood ratio**  **(G^2^) test** | | |
| --- | --- | --- | --- | --- | --- |
|  |  |  | **Chi^2^_ms (modelvs.saturated)** | **p>chi^2^** | |
| 1 | 40149.04 | 40248.55 | 5252.53 | | 1.000 |
| 2 | 38869.56 | 39074.8 | 3939.05 | | 1.000 |
| 3 | 38590.49 | 38901.46 | 3625.99 | | 1.000 |
| 4 | 38512.27 | 38928.96 | 3513.76 | | 1.000 |
| 5 | 38457.06 | 38967.05 | 3428.56 | | 1.000 |

AIC: Akaike information criterion. BIC: Bayes information criterion
