# Supplementary material for: Latent class analyses of multimorbidity and all-cause mortality: A prospective study in Chilean adults
Source: PLoS One. 2023 Dec 19;18(12):e0295958. doi: 10.1371/journal.pone.0295958 (PMC10729966; doi:10.1371/journal.pone.0295958)
Supplement: S1 Fig — (DOCX) [file pone.0295958.s002.docx]

**Participants in the CHNS 2009-2010**

N= 5,293

Participants included in the final analysis

N= 3,701

**Exclusion**

Participants without data for all covariates and follow-up N= 134

Participants with available data for Chronic Conditions

N= 3,835

**Exclusion**

Participants without data for chronic conditions, N= 1,458

# **S1 Fig. Participants included in the analysis (electronic version)**
