# Supplementary figures and images for: Latent class analyses of multimorbidity and all-cause mortality: A prospective study in Chilean adults
Source: PLoS One. 2023 Dec 19;18(12):e0295958. doi: 10.1371/journal.pone.0295958 (PMC10729966; doi:10.1371/journal.pone.0295958)

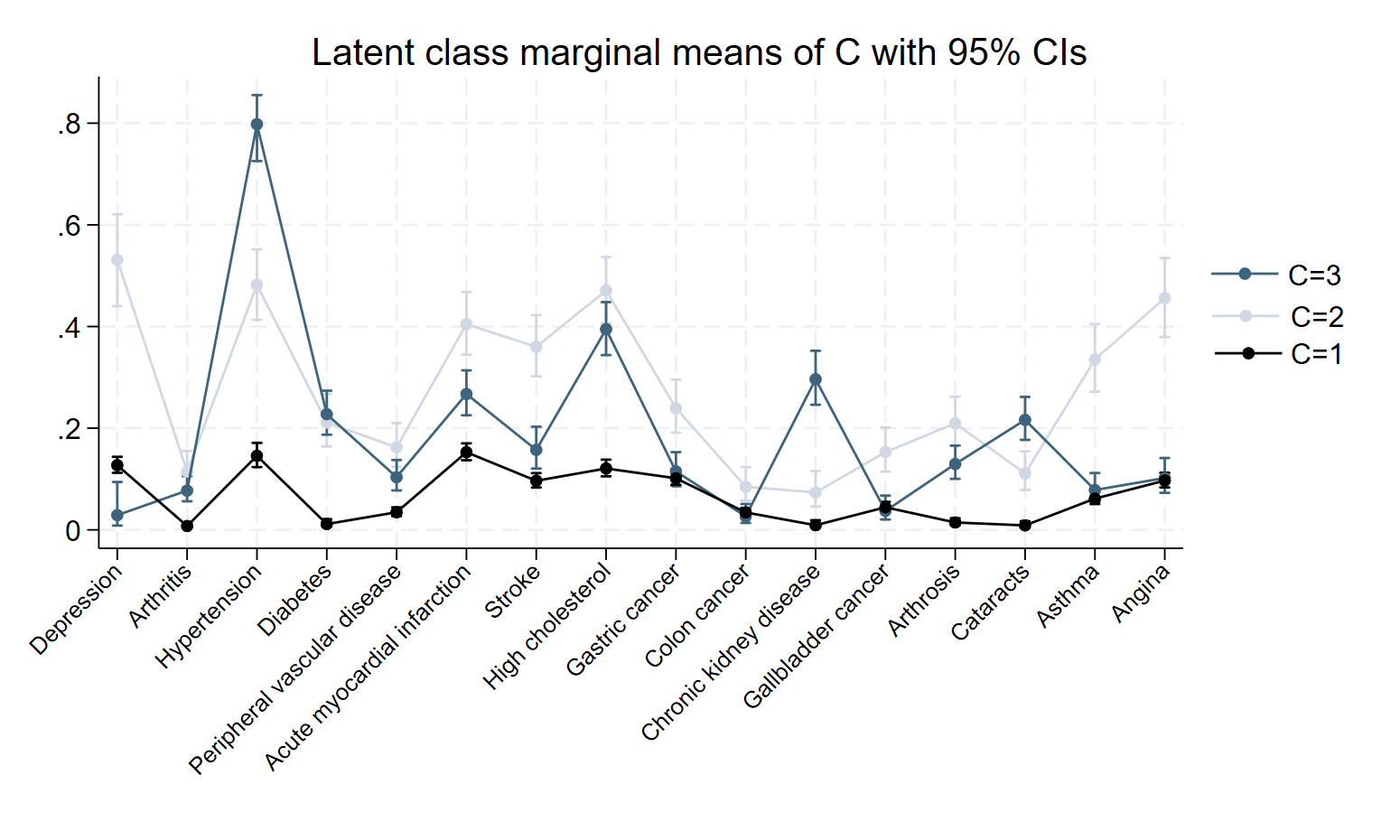


# **S2 Fig. Latent Classes identified**

Supplement: S2 Fig — (DOCX) [file pone.0295958.s003.docx]
